# Microcytic Anemia Hiding Vitamin B12 Deficiency Anemia

**DOI:** 10.7759/cureus.20741

**Published:** 2021-12-27

**Authors:** Fadi Busaleh, Omkolthoom A Alasmakh, Fatimah Almohammedsaleh, Maram F Almutairi, Juwdaa S Al Najjar, Abbas Alabdulatif

**Affiliations:** 1 Pediatrics, Maternity and Children Hospital, Ministry of Health, Al-Ahsa, SAU; 2 Ophthalmology, Al Jabr Hospital, Ministry of Health, Al-Ahsa, SAU; 3 Internal Medicine, King Fahad General Hospital, Ministry of Health, Al-Ahsa, SAU; 4 Family Medicine, National Guard Health Affairs, Riyadh, SAU; 5 Medicine, College of Medicine, King Faisal University, Al-Ahsa, SAU; 6 Pediatric Hematology/Oncology, Maternity and Children Hospital, Ministry of Health, Al-Ahsa, SAU

**Keywords:** cobalamin, microcytic anemia, macrocytic anemia, anemia, vitamin b12

## Abstract

Vitamin B12 is an essential water-soluble vitamin that mediates multiple coenzymes needed for cell synthesis, mainly the red blood cells. Its deficiency is characterized by megaloblastic anemia and neuropsychiatric symptoms. Macrocytosis is the classical picture seen usually, but having microcytosis is unlikely. We report a case series of three cousins with vitamin B12 deficiency who presented with microcytosis.

## Introduction

Anemia is a major health problem in the world, with variable etiologies. It is defined as a drop in hemoglobin level two standard deviations of normal range for both age and gender. As result, there will be a decrease in oxygen-carrying capacity, which can lead to tissue hypoxia [1]. Anemia is divided according to the red blood cell (RBC) sizes, which is called mean corpuscular volume, into three categories: microcytic anemia, normocytic anemia, and macrocytic anemia [1,2]. The most common cause of anemia worldwide is iron deficiency, which results in microcytic hypochromic anemia [2]. Sometimes, there is a concurrent presence of different types of anemia, which result in masking the clinical findings of one of them [3]. We report a case series of three cousins who had microcytic anemia as a masking sign of vitamin B12 deficiency.

## Case presentation

Case 1

A two-year-old Saudi boy, with non-significant medical history, presented to the Pediatric Emergency Department at Maternity and Children Hospital in Al-Ahsa, Eastern Providence, Saudi Arabia, with a history of intermittent recurrent vomiting, pallor, and lethargy. The vomiting started three months back, which was intermittent, non-projectile with gastric juice content with no diurnal variation, but it was associated with a decrease in appetite and weight loss of around 5 kg. It was not associated with jaundice, fever, or any abnormal movement. There were recurrent ER visits among the past three months, which were managed by anti-emetics. At the end, vomit become more frequent, and the child became dehydrated with no response to anti-emetics for one week; thus, the family sought medical advice again. The patient was admitted to the pediatric ward as the patient was dehydrated and needed further investigations due to persistence vomiting to rule out the suspension of increased intracranial pressure due to a space-occupying lesion.

Physical examination showed a pale, hypoactive, underweight child, and vital signs showed mild tachycardia (146 beats/minutes) and maintained blood pressure of 82/50 mmHg. His weight was 9.8 kg (just on the fifth centile for age). Cardiac examination showed mid-systolic hemic murmur, with unremarkable rest examination.

**Figure 1:**
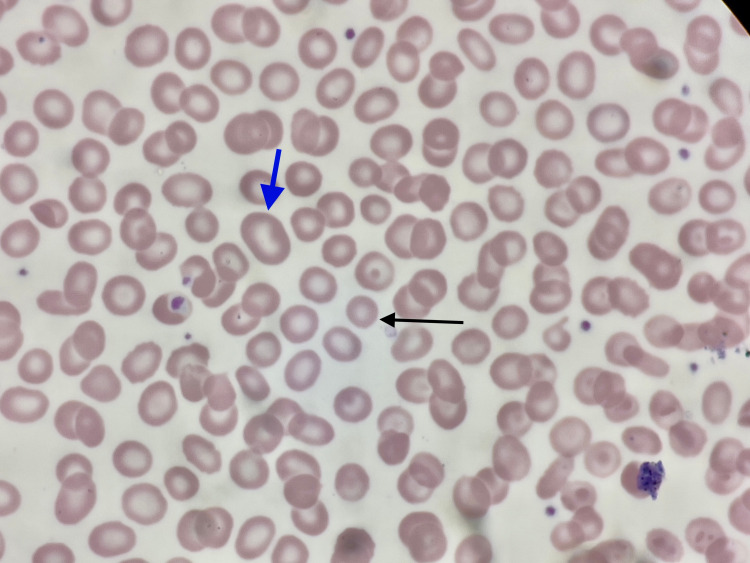
Peripheral blood film (microscopic view) showing a mixed picture of macrocytosis (blue arrow) and microcytosis (black arrow)

Initial laboratory tests were significant for microcytic hypochromic anemia (Table 1). Peripheral blood smear showed a mixed picture of microcytic and macrocytic RBC morphology (Figure 1). Computed tomography scans of the brain and abdominal ultrasound were performed, and they were unremarkable.

**Table 1:** Laboratory findings in the three patients

Test	Patient 1	Patient 2	Patient 3	Reference range
Complete blood count				
White blood cell count	13.57	27.78	21.59	3-14x10^3 ^/uL
Red blood cells count	3.48	2.16	3.62	4.2-6.1 x10^6^/uL
Hemoglobin	6.8	4.1	10	11.1-12.6 g/dL
Platelets	162	109	372	150-350x10^3^/uL
Mean corpuscular volume	66	61.6	63.6	70-78 fL
Reticulocyte count	2.5%	0.31%	-	05-1%
Blood chemistry tests				
Iron level	27	9.5	8.3	9-31.3 micmol/L
Total iron-binding capacity	50.85	-	60	44.75-80.55 micmol/L
Hematological workup				
Hemoglobin A1 level (HbA1)	92.2%	92%	92.4 %	95-98%
Hemoglobin A2 level (HbA2)	3%	5%	4.0 %	<2.2%
Fetal hemoglobin level (HbF)	4.8%	3%	3.6 %	<0.5%
Hemoglobin H preparation	Negative	Negative	Negative	Negative
Vitamin B12 level	19	50	34	200-900 pg/mL

As there was a family history of vitamin B12 deficiency with a similar presentation, a vitamin B12 level was requested. The result came to be low (36 pg/mL) (Table 1), and that explained the megaloblastic anemia and the presenting symptoms secondary to vitamin B12 deficiency. Concurrent presence of thalassemia trait (high HbA2 levels of 4.8%) may be the reason that masked the megaloblastic anemia.

The patient was managed with packed RBCs transfusion initially for restoration of a safe hemoglobin level and continued on vitamin B12 0.5 mg intramuscularly (IM) monthly with regular follow-up with a pediatric hematologist.

Case 2 

**Figure 2:**
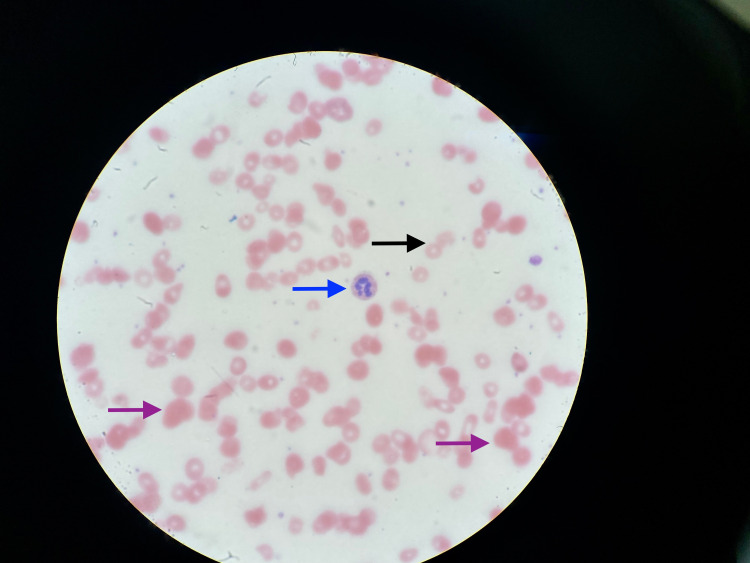
Peripheral blood film (microscopic view) showing a mixed picture of microcytosis (black arrow), macrocytosis (purple arrows), and hypersegmented neutrophils with six nuclei.

A 20-month-old girl, with non-significant medical history, presented to the Pediatric Emergency Department at Maternity and Children Hospital with a history of high-grade fever for 10 days, continuous, and unremitting, with no response to antibiotic or antipyretics. There was no history of cough, vomiting, diarrhea, jaundice, or any changes in behavior or abnormal movements. Upon examination, she was pale, lethargic, and irritable. Her vital signs were as follows: temperature of 38.5°C febrile and tachycardic (150 beats/minute) with a prolonged capillary filling time of >4 seconds with mottled skin. Cardiovascular examination revealed a mid-systolic hemic murmur grade 3/6 in the left upper sternal border with no radiations, and the rest of the examination was unremarkable. Her investigations showed pancytopenia with positive pus cells 10 in urine analysis (Table 1). She was admitted to the pediatric intensive care unit as a case of septic shock secondary to urinary tract infection to rule out bone marrow malignancies. Blood and urine cultures were taken, and she was started on empiric antibiotics and received a blood transfusion to improve her hemodynamic state with normal saline boluses. Peripheral blood smear showed no blasts cells, but there was a mixed picture of microcytic and macrocytic on RBCs morphology with hypersegmented neutrophils (Figure 2).

As the mother mentioned a history of prolonged pallor with no response to iron supplementation and a family history of vitamin B12 deficiency in one of her cousins, vitamin B12 level was requested to rule out its deficiency.

The level of vitamin B12 came low (50 pg/mL) (Table 1). Therefore, the patient was diagnosed with vitamin B12 deficiency with exacerbation secondary to urinary tract infection. Also, hemoglobin electrophoresis was performed, which showed a high HbA2 level (3 %) with normal other parameters suggestive for the thalassemia trait.

The patient was started on vitamin B12 1,000 mcg IM daily for seven days and then 100 mcg IM monthly, with regular follow-up with a pediatric hematologist.

Case 3 

A 20-month-old Saudi boy, with a non-significant medical history, presented with a history of pallor, irritability, lethargy, drooling of saliva, decreased appetite, and weight loss for six months. The patient was seen at primary health care initially and was found to have microcytic hypochromic anemia with a low iron profile.

He was started on oral iron supplements for one month with no improvement. After that, the patient was referred to the Pediatric Hematology Center for Hereditary Diseases in Al-Ahsa for further investigation and management. At the Pediatric Hematology Center for Hereditary Diseases, the pediatric hematologist found that the patient has a family history of vitamin B12 deficiency with a similar presentation in his older sister.

**Figure 3:**
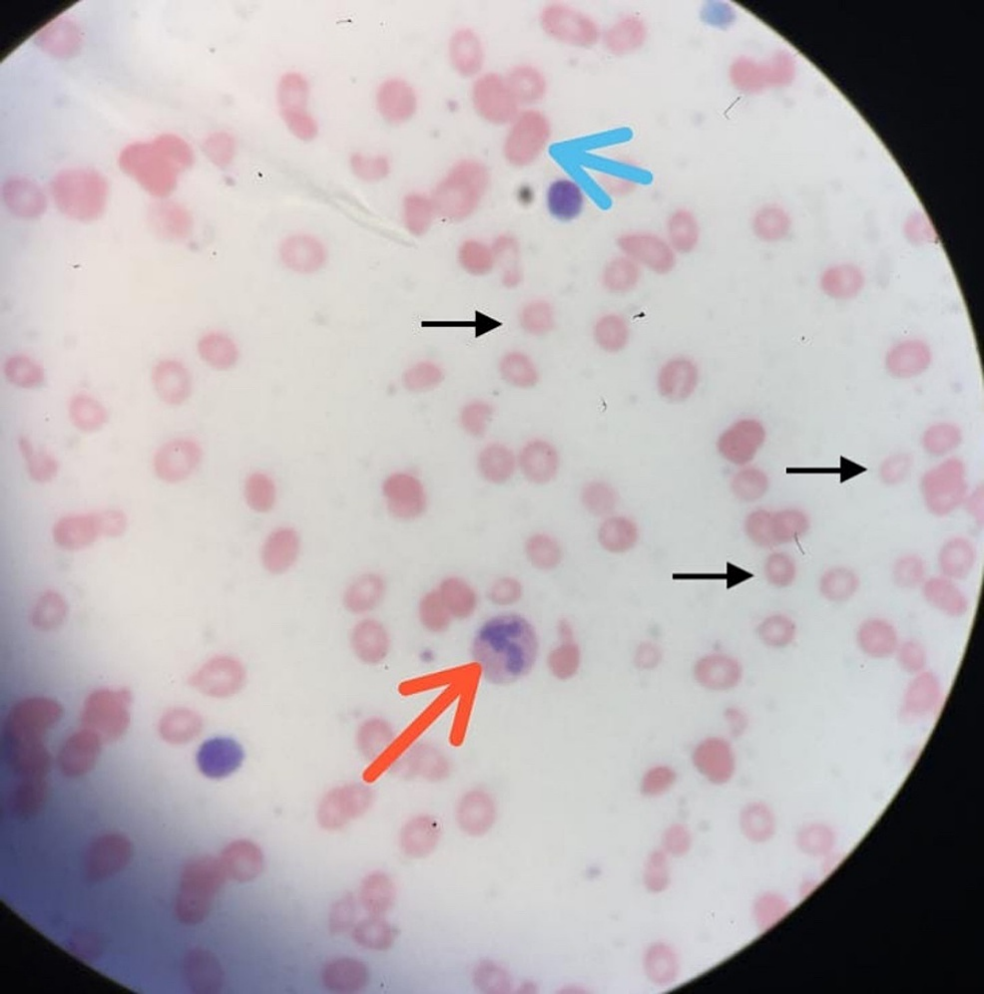
Peripheral blood film (microscopic view) showing a mixed picture of microcytosis (black arrow), macro-ovalocyte (blue arrow), and hypersegmented neutrophils (red arrow).

His investigations showed microcytic anemia in complete blood count (Table 1). Peripheral blood smear and vitamin B12 levels were requested. The peripheral blood smear showed a mixed picture of microcytic hypochromic anemia in addition to macrocytosis with hypersegmented neutrophils (Figure 3). Vitamin B12 level was 34 pg/mL, which is low (Table 1). Hemoglobin electrophoresis was also performed, which showed a high HbA2 level (3.69 %) with normal other parameters suggestive for the thalassemia trait. The patient was diagnosed with vitamin B12 deficiency and started on vitamin B12 0.5 mg IM monthly with folic acid 1 mg orally once daily and he responded well.

## Discussion

Cobalamin or vitamin B12 is a water-soluble vitamin. It is present in animal sources such as red meat, liver, kidney, or dairy products. Absorption of it is mediated by the intrinsic factor (IF) secreted from the stomach to combine to it and form IF complex, which mediated the final absorption step in the terminal ileum. Vitamin B12 has an essential role in the formation of crucial proteins and DNA or RNA synthesis needed for the formation of hematopoietic cells. This essential metabolic process depends on the synthesis of the byproduct or active metabolites from cobalamin, which are methylcobalamin and adenosylcobalamin [4,5 ].

Vitamin B12 deficiency is caused by different etiologies. First, nutritional deficiency of vitamin B12 is seen with vegans who strictly do not consume animal products. An example of such in pediatrics is seen with infants born to vegans’ mothers or a mother with pernicious anemia who has a deficiency of IF. Secondly, vitamin B12 deficiency is also related to impaired absorption. This category may be related to a defect in IF as in the case of pernicious anemia with autoimmune gastritis or defect in the terminal ilium as the final place of absorption of vitamin B12 [6,7].

Clinical manifestations are variable, nonspecific symptoms of which are related to anemia indeed, such as weakness, lethargy, headache, and pallor. Some gastrointestinal symptoms can also be seen, such as feeding difficulties, drooling of saliva, failure to thrive, diarrhea, and vomiting and glossitis secondary to megaloblastic anemia [5]. Neurological manifestations are the main concern as the child may show growth delay or regression of growth. Cognitive impairment and irritability are also widely seen. The most significant symptom seen is symmetrical paresthesia and numbness, which affect the lower limbs more than the upper one with ataxia. If left untreated or unnoticed, this can progress to motor impairment and paraplegia [4,5]. Most of the nonspecific symptoms were seen in our patients. Regarding the neurological symptoms seen in our patient were irritability and some behavioral changes, with no weakness observed. The absence of advance neurological symptoms is related to the presence of index case that aid in early diagnosis and hence fast management.

Diagnosis of vitamin B12 deficiency is made by a constellation of symptoms and integration of relevant investigations. Drop in hemoglobin and macrocytosis is the classical picture seen in the initial hematological workup. Sometimes, there is pancytopenia and hypersegmented neutrophils associated with megaloblastic anemia. Associated low levels of vitamin B12 and its metabolites is also there [8-10]. In comparison to our patients, they had low levels of vitamin B12 but microcytic anemia, which may be related due to the coinheritance of thalassemia as our area is enriched with such cases. This is supported by the high levels of HbA2 levels, mainly beta-thalassemia, as there is a negative Hb H preparation test, which makes alfa thalassemia unlikely. Unfortunately, genetic studies are needed, but it is not available in our center to confirm the type of thalassemic association and determine if there are genetic factors involved in this coincidence of vitamin B12 deficiencies among different cousins of the same family [11,12]. Iron deficiency anemia can be associated with vitamin B12 deficiency but this is excluded as the iron levels were normal.

Management of vitamin B12 deficiency is directed according to the cause. In case of nutritional deficiency, oral supplementation can be effective alone. On the other hand, in case of absorption impairment, parenteral routes of vitamin B12 administration are the appropriate choice [13]. As our patients' causes for vitamin B12 deficiency seems to be familial and the history was not suggestive of nutritional causes, vitamin B12 IM every month was chosen for management.

## Conclusions

Differentiation between different types of anemia is determined by the volume of RBCs, which aid in the identification of the causative reason for anemia. Classical medical teaching tells that macrocytic anemia is caused by either vitamin B12 or folate deficiency but not microcytosis. Microcytosis seen in our case report masks the early presentation of megaloblastic anemia and deceives physicians. This should alert the physician to investigate concurrent deferential causes of anemia and think broadly when encountering cases that cannot be explained by one differential diagnosis.
